# Prevalence of Venous Thromboembolism in Critically Ill COVID-19 Patients: Systematic Review and Meta-Analysis

**DOI:** 10.3389/fcvm.2020.598846

**Published:** 2021-01-08

**Authors:** Mouhand F. H. Mohamed, Shaikha D. Al-Shokri, Khaled M. Shunnar, Sara F. Mohamed, Mostafa S. Najim, Shahd I. Ibrahim, Hazem Elewa, Lina O. Abdalla, Ahmed El-Bardissy, Mohamed Nabil Elshafei, Ibrahim Y. Abubeker, Mohammed Danjuma, Khalid M. Dousa, Mohamed A. Yassin

**Affiliations:** ^1^Department of Medicine, Hamad Medical Corporation, Doha, Qatar; ^2^College of Pharmacy, QU Health, Qatar University, Doha, Qatar; ^3^Clinical Pharmacy Department, Hamad General Hospital, Doha, Qatar; ^4^Alpert Medical School, Brown University, Providence, RI, United States; ^5^College of Medicine, QU Health, Qatar University, Doha, Qatar; ^6^Division of Infectious Diseases and HIV Medicine, University Hospitals Cleveland Medical Center, Case Western Reserve University, Cleveland, OH, United States; ^7^Department of Hematology, Hamad Medical Corporation, Doha, Qatar

**Keywords:** COVID-19, SARS-CoV-2, VTE, thrombosis, venous, ICU, DVT—deep vein thrombosis

## Abstract

**Background:** Recent studies revealed a high prevalence of venous thromboembolism (VTE) events in coronavirus disease 2019 (COVID-19) patients, especially in those who are critically ill. Available studies report varying prevalence rates. Hence, the exact prevalence remains uncertain. Moreover, there is an ongoing debate regarding the appropriate dosage of thromboprophylaxis.

**Methods:** We performed a systematic review and proportion meta-analysis following the Preferred Reporting Items for Systematic Reviews and Meta-Analyses (PRISMA) guidelines. We searched PubMed and EMBASE for studies exploring the prevalence of VTE in critically ill COVID-19 patients till 25/07/2020. We pooled the proportion of VTE. Additionally, in a subgroup analysis, we pooled VTE events detected by systematic screening. Finally, in an exploratory analysis, we compared the odds of VTE in patients on prophylactic compared with therapeutic anticoagulation.

**Results:** The review comprised 24 studies and over 2,500 patients. The pooled proportion of VTE prevalence was 0.31 [95% confidence interval (CI) 0.24, 0.39; *I*^2^ 94%], of VTE utilizing systematic screening was 0.48 (95% CI 0.33, 0.63; *I*^2^ 91%), of deep venous thrombosis was 0.23 (95% CI 0.14, 0.32; *I*^2^ 96%), and of pulmonary embolism was 0.14 (95% CI 0.09, 0.20; *I*^2^ 90%). Exploratory analysis of few studies, utilizing systematic screening, VTE risk increased significantly with prophylactic, compared with therapeutic anticoagulation [odds ratio (OR) 5.45; 95% CI 1.90, 15.57; *I*^2^ 0%].

**Discussion:** Our review revealed a high prevalence of VTE in critically ill COVID-19 patients. Almost 50% of patients had VTE detected by systematic screening. Higher thromboprophylaxis dosages may reduce VTE burden in this patient's cohort compared with standard prophylactic anticoagulation; however, this is to be ascertained by ongoing randomized controlled trials.

## Introduction

The pool of recent evidence suggests that coronavirus disease 2019 (COVID-19) is a thrombogenic condition. It leads to an increased incidence of both venous and arterial thromboembolic events (1). COVID-19 patients admitted to the intensive care units (ICU) seem to carry a higher risk (1). Venous thromboembolism (VTE) prevalence in the critically ill COVID-19 patients varied across individual studies. This is likely due to differences in screening methods (systematic vs. non-systematic screening), among other study-specific characteristics, leaving VTE's exact prevalence unknown. The prevalence of deep venous thrombosis (DVT) was considered low compared with pulmonary embolism (PE), which led researchers to consider microthrombosis as an additional mechanism of PE in COVID-19 patients (2).

VTE's heightened risk led to a wide chemoprophylaxis use for critically ill COVID-19 patients (3). Notwithstanding this, recent studies showed that even COVID-19 patients on chemoprophylaxis remain to carry a high risk of VTE compared with non-COVID-19 patients (4). As a result, guidance driven by expert opinions suggested utilizing higher doses of anticoagulation (1). However, this recommendation lacks robust, supporting systematic studies. Thus, we aimed to systematically review the literature and explore the pooled prevalence of VTE, PE, and DVT in critically ill COVID-19 patients. Additionally, we aimed to evaluate the yield of systematic VTE screening and its effect on the prevalence. Moreover, if data allow, we aimed to examine the odds of VTE in patients on prophylactic compared with therapeutic anticoagulation.

This review follows the Preferred Reporting Items for Systematic Reviews and Meta-Analyses (PRISMA) guidelines (5). It is pre-registered at the International Prospective Register of Systematic Reviews (PROSPERO) (registration number: CRD42020185916).

## Eligibility Criteria

We limited our review to observational studies (cohort, cross-sectional, retrospective, or case series), estimating the proportion of VTE events in critically ill COVID-19 adult (>18 years) patients (admitted to the ICU). To facilitate a timely review, we limited our inclusion to articles written in the English language only. We excluded studies where the proportion of VTE could not be ascertained or if the population of interest is not ICU patients.

## Information Sources and Literature Search

For a timely review, we performed the search in PubMed, MEDLINE, and EMBASE. We used free text, emtree, and MeSH terms in our search. There were no language or date limitations implied in the search. The last date of the formal search was the 10th of July 2020; however, we performed a scoping search till the 25th of July 2020. Example of a utilized search strategy was [(“venous thromboembolism” OR “deep vein thrombosis” OR “lung embolism” OR “vein thrombosis”/exp/mj) AND [embase]/lim] AND [(“covid 19” OR (coronavirus AND disease AND 2019) OR (sars AND cov AND 2) OR “covid 19”/exp/mj) AND [embase]/lim]. We also performed relevant citations and reference searches.

## Screening and Data Extraction

Two reviewers (MM and SM) conducted the screening in two stages. The first stage was screening the retrieved articles' titles and abstracts independently. Secondly, the articles' full text was retrieved and assessed for inclusion. When disagreement occurred, a third reviewer (LA) settled the disagreement guided by the protocol. We used pre-made excel sheets to collect relevant articles data. This included the last author name, publication date, study country, sample size, events number (DVT, PE, and VTE), baseline characteristics (median age, gender frequency, average BMI, and other comorbidities), intubation frequency, thromboprophylaxis frequency, and follow-up duration.

## Study Quality and Risk of Bias Assessment

We used a validated tool for assessing the risk of bias of prevalence studies. The tool was devised by Hoy et al. and is composed of 10 items summarizing four domains (6). We additionally generated funnel plots to examine the risk of publication bias in our review.

## Data Analysis

A scoping review revealed heterogeneity of the method of VTE screening, reporting, and detection. Additionally, there were varying follow-ups given the nature of ICU admitted patients. Hence, neither the true incidence (different follow-up times and some patients may already have the event of interest before the study) nor the true prevalence (varying follow-up times and absence of unifying screening for all individuals at risk) could be accurately pooled. We instead decided *a priori* to pool a proportion of VTE with a 95% confidence interval (CI). This proportion represents the number of patients with the event of interest divided by the study population at risk during the study regardless of their follow-up duration. We felt that this would be a proxy or an estimate of the prevalence. We used the validated method of double arcsine transformation to stabilize the variance and confine the CI between 0 and 1 (7). We generated forest plots to display the results of the analysis. We used the Cochrane Q test and *I*^2^ to examine heterogeneity. *I*^2^ >60% indicates significant heterogeneity. Regardless of the heterogeneity, we would use the random-effects model (REM) in our analysis. We used MetaXl software for statistical analysis (version 5.3©, EpiGear International Pty Ltd., ABN 51 134 897 411, Sunrise Beach, Queensland, Australia, 2011–2016).

## Subgroup and Sensitivity Analyses

We *a priori* decided to examine the proportion of DVT and PE. Additionally, we looked at the proportion of VTE in various populations (systematic screening vs. non-systematic screening, therapeutic vs. prophylactic anticoagulant dose). Moreover, we performed a sensitivity analysis to reflect the relative constituent studies' impact on the consistency of the pooled proportion of the primary endpoint.

## Results

### Included Studies and Baseline Characteristics

**Figure 1:**
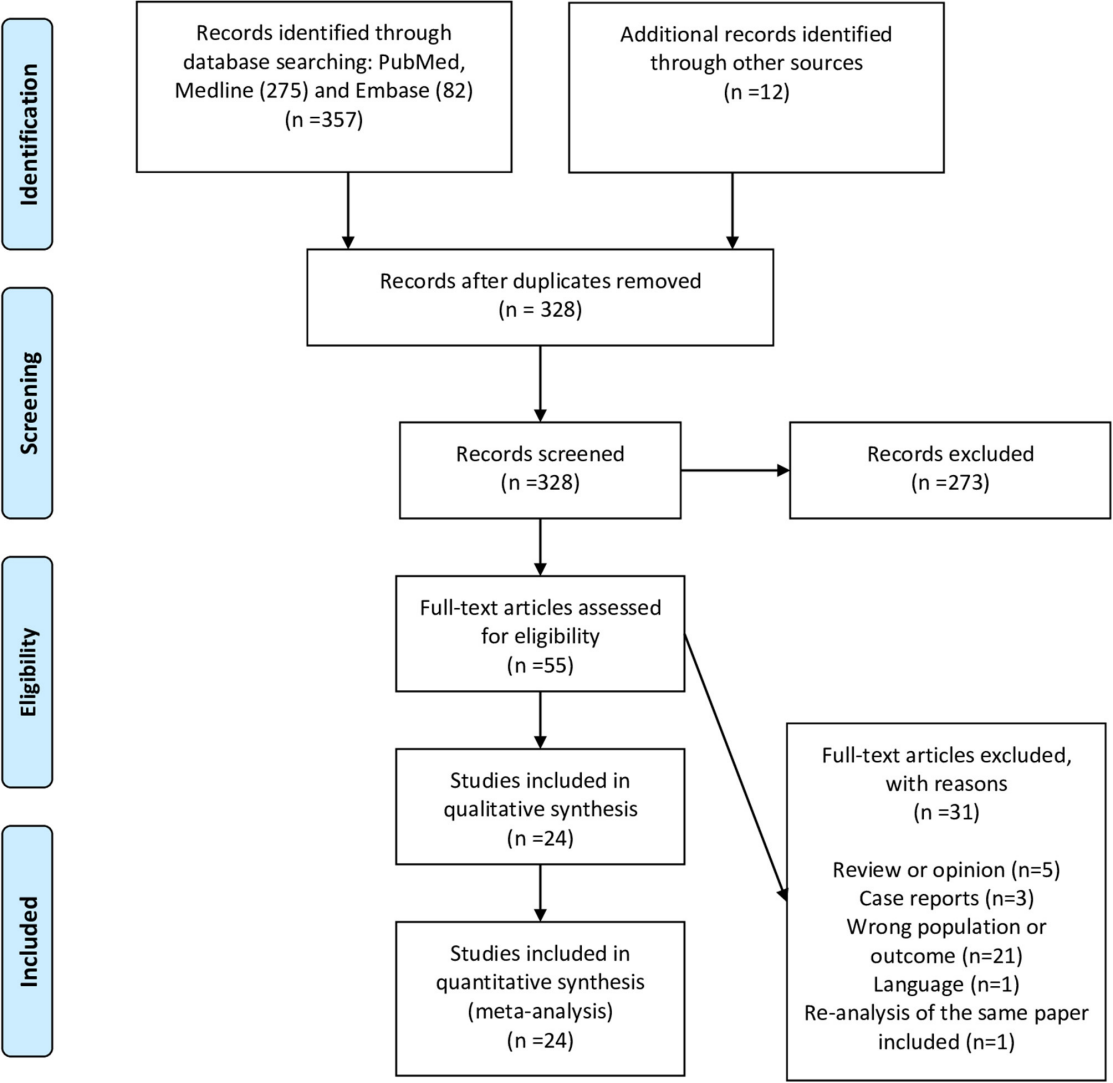
PRISMA flow diagram.

Twenty-four studies describing a total of 2,570 patients were included in our final analysis (Figure 1 shows the flow diagram) (4, 8–29). The studies were heterogeneous in terms of VTE events identification and screening (Table 1). In 10 studies, the screening for VTE was systematically done using lower and upper limb ultrasound (US) (systematic screening was only for DVT and not PE). Fourteen studies evaluated for the presence of VTE based on clinical suspicion and further confirmation by imaging (non-systematic). Twenty-two studies reported the proportion of DVTs, and 17 studies reported the proportion of PE events. Out of the 10 studies where systematic screening was adopted, the screening was incomplete in one. In all studies but one (25), most patients were on thromboprophylaxis with varying doses.

**Table 1:** Summary of included studies.

**Study (location)**	**Study design**	**Study duration in days**	**Total number**	**Age, mean, or median (males percentage %)**	**Intubated %**	**D-dimers (mean or median)**	**Pharmacologic prophylaxis %**	**Screening method**	**VTE proportion % (numbers)**	**Mortality %**
Al-Samkari et al. (United States) (8)	Retrospective analysis	36 days (March–April 2020)	–	65 (males 64.7%)	–	–	98.6% (12.5% intermediate or full anticoagulation)	Clinical suspicion	10.4% (15/144)	18.8% (27/144)
Beun et al. (Netherlands) (19)	Retrospective analysis	24 days (March–April 2020)	75	–	–	–		Clinical suspicion	30.6% (23/75)	–
Bilaloglu et al. (United States)(23)	Retrospective analysis	48 days (March–April 2020)	829	–	–	–	Most patients (percentage not specified)	Clinical suspicion	13.6% (113/829)	54.4% (451/829)
Criel et al. (Belgium) (24)	Retrospective analysis	24 days (April 2020)	30	64.5 (males 67%)	70%	1,400 ng/ml	100% (intermediate prophylactic dose)	Systematic screening (Doppler US of upper and lower limbs)	13.3% (4/30)	13.3% (4/30)
Cui et al. (China) (25)	Retrospective analysis	53 days (Jan–March 2020)	81	59.9 (males 46%)	–	5,200 ng/ml	0%	Systematic screening (lower limb Doppler US)	24.6% (20/81)	10% (8/81)
Desborough et al. (United Kingdom) (26)	Retrospective analysis	31 days (March 2020)	66	59 (males 73%)	79%	1,200 ng/ml	100% (83% prophylactic, 17% therapeutic)	Clinical suspicion	16.6% (11/66)	30.3% (20/66)
Fraissé et al. (France) (27)	Retrospective analysis	–	92	61 (males 79%)	89%	2,400 ng/ml	100% (47% prophylactic, 53% therapeutic)	Clinical suspicion	33.6% (31/92)	–
Grandmaison et al. (Switzerland) (28)	Retrospective analysis	–	29	66 (males 64.7%)	–	8,760 ng/ml	93% (96% prophylactic, 4% therapeutic)	Systematic screening (Doppler US of upper and lower limbs)	58.6% (17/29)	–
Helms et al. (France) (29)	Retrospective analysis	29 days (March 2020)	150	63 (males 81%)	100%	2,270 ng/ml	100% (70% prophylactic, 30% therapeutic)	Clinical suspicion	18.6% (28/150)	8.70% (13/150)
Hippensteel et al. (United States) (9)	Retrospective analysis	28 days (March–April 2020)	91	55 (males 57%)	85%	1,071 ng/ml	54.3% therapeutic	Clinical suspicion	26.3% (24/91)	22% (22/91)
Klok et al. (Netherlands) (10)	Retrospective analysis	47 days (March–April 2020)	184	64 (males 76%)	–	–	100% (90.8% prophylactic, 9.2% therapeutic)	Clinical suspicion	36.9% (68/184)	22% (41/184)
Llitjos et al. (France) (4)	Retrospective analysis	24 days (March–April 2020)	26	68 (males 77%)	100%	1,750 ng/ml	100% (prophylactic 31%, therapeutic 69%)	Systematic screening (compression and Doppler US)	69.2% (18/26)	12% (3/26)
Lodigiani et al. (Italy) (11)	Retrospective analysis	58 days (February–April 2020)	48	61 (males 80.3%)	–	615 ng/ml	100% (40% weight adjusted or therapeutic)	Clinical suspicion	8.3% (4/48)	–
longchamp et al. (Switzerland) (12)	Retrospective analysis	26 days (March–April 2020)	25	68 (males 64%)	92%	2,071 ng/ml (953–3,606)	100% (prophylactic 23/25, therapeutic 2/25)	Systematic screening (proximal lower extremity DVT)	32% (8/25)	20% (5/25)
Maatman et al. (United States) (13)	Retrospective analysis	20 days (March 2020)	109	61 (males 57%)	94%	84,506 ng/ml	100% (prophylactic 102/109, therapeutic 7/109)	Clinical suspicion	28.4% (31/109)	25% (27/109)
Middeldorp et al. (Netherlands) (14)	Retrospective analysis	42 days (March–April 2020)	75	62 (males 58%)	100%	2,000 ng/ml	100%	Systematic screening (lower limb Doppler every 5 days)	46.6% (35/75)	
Moll et al. (United States) (15)	Retrospective analysis	38 days (March–April 2020)	102	64.61 (males 57.8%)	86.3%	3,964 ng/ml	97.1% (89.8% prophylactic, 10.1% therapeutic)	Clinical suspicion	8.8% (9/102)	27.5% (28/102)
Nahum et al. (France) (16)	Case series	Mid-March–April 2020	34	62.2 (males 78%)	100%	27,927 ng/ml	100% prophylactic anticoagulation	Systematic screening (lower limbs US for all patients)	79.4% (27/34)	Not mentioned
Pineton De Chambrun et al. (France) (17)	Retrospective analysis	26 days (March–April 2020)	25	47.7 (males 68%)	–	Highly elevated (NS)	100% therapeutic	Clinical suspicion	24% (6/25)	–
Poissy et al. (France) (18)	Retrospective analysis	34 days (February–March 2020)	107	57 (males 59%)	62.6%	–	100%	Clinical suspicion	22.4% (24/107)	14% (15/107)
Ren et al. (China) (22)	Cross-sectional	3 days (Feb–March)	48	70 (males 54.2%)	37.5%	3,480 ng/ml	97.9% prophylactic	Systematic screening (proximal and distal lower limbs compression US)	85.4% (41/48)	31.3% (15/48)
Stessel et al. (Belgium) (20)	Quasi-experimental	18 days (March 2020)	46	69.5 (males 73.9%)	–	970 ng/ml	100% Prophylactic standard dose	Systematic screening	41.3% (19/46)	39.13% (18/46)
Stessel et al. (Belgium) (20)	Quasi-experimental	21 days (March–April 2020)	26	62 (males 57.3%)	–	2,180 ng/ml	100% Intensive prophylactic dose	Systematic screening (Doppler US and compression US of the great veins in upper and lower limbs)	15.3% (4/26)	3.85% (1/26)
Thomas et al. (United Kingdom) (21)	Retrospective analysis	33 days (March–April 2020)	63	59 (males 69%)	83%	394 ng/ml	100% (prophylactic dose)	Clinical suspicion	9.5% (6/63)	8% (5/63)
Zhang et al. (China) (22)	Retrospective analysis	32 days (January February 2020)	65	–	–	–	–	Systematic screening (lower limbs US Doppler for DVT at proximal and distal levels)	66.1% (43/65)	–

*(–) Refers to data unavailable for the ICU cohort*.

## The Proportion of VTE Events

**Figure 2:**
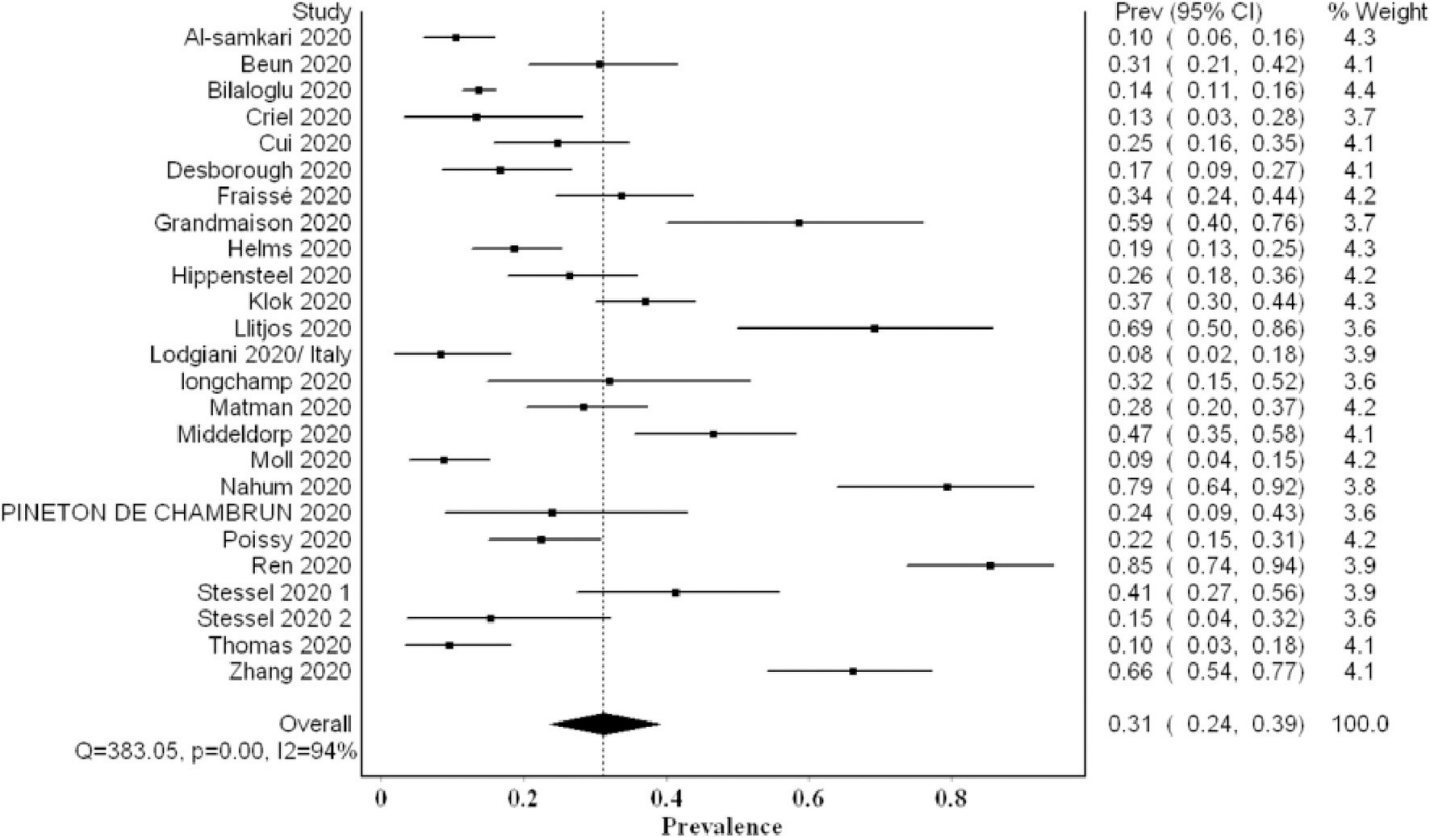
Forest plot showing the overall pooled proportion of VTE events.

The overall pooled proportion of VTE from 24 studies examining a total of 2,570 was 0.31 (95% CI 0.24, 0.39; *I*^2^ 94%; Q 383) with significant heterogeneity (Figure 2). The funnel plot showed significant asymmetry suggestive of possible publication bias (Supplementary 1). The sensitivity analysis did not affect the final point estimate significantly (Supplementary 2).

## The Proportion of VTE Utilizing Systematic Screening

**Figure 3:**
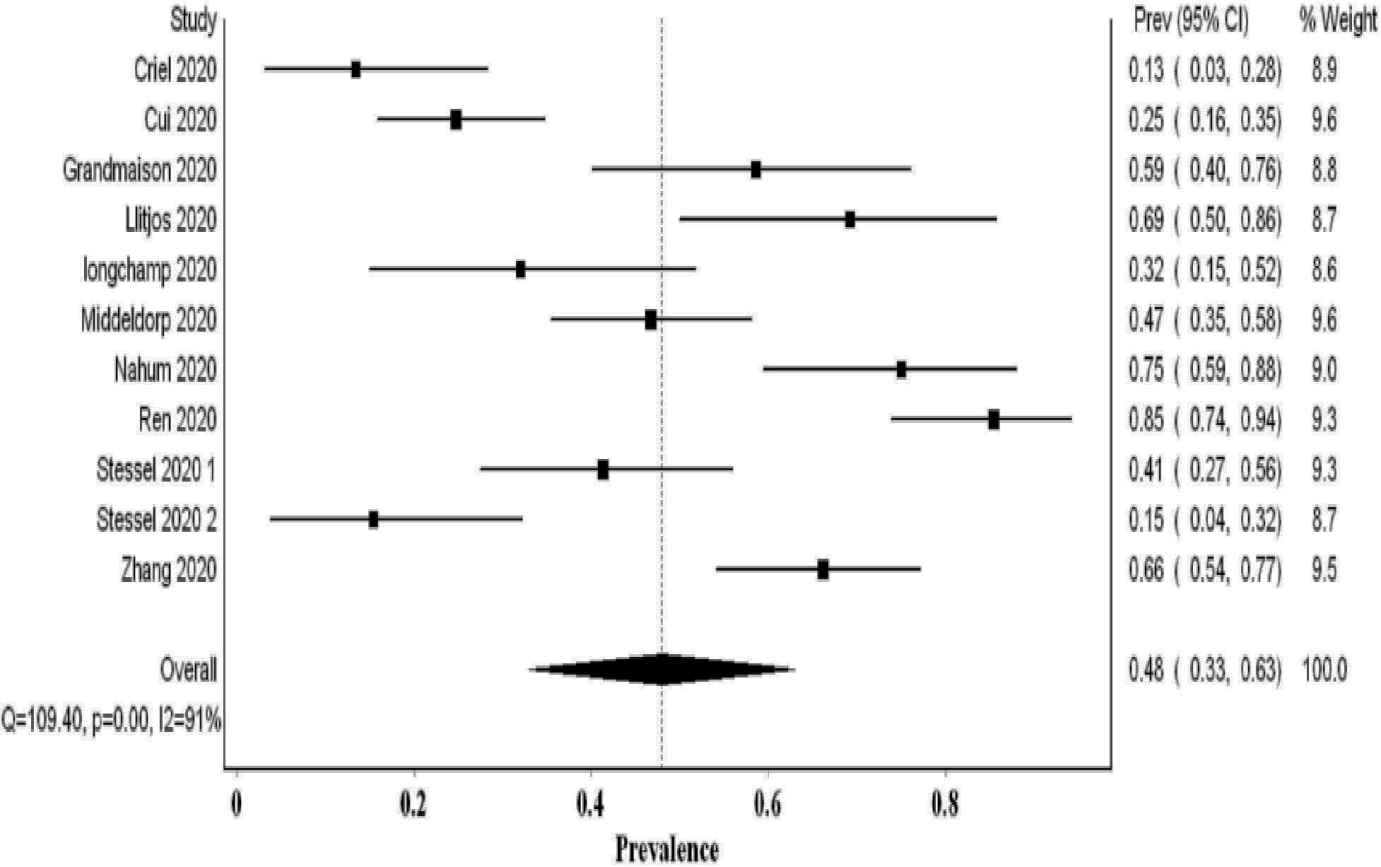
Forest plot showing the pooled proportion of VTE events utilizing systematic screening methods.

Ten studies examining 478 patients using systematic screening revealed a higher VTE proportion of 0.48 (95% CI 0.33, 0.63; *I*^2^ 91%; Q 109) with significant heterogeneity (Figure 3). The funnel plot suggested a publication bias (Supplementary 3). The exclusion of Cui et al.'s study that did not utilize thromboprophylaxis resulted in a higher proportion of VTE events of 0.51. Additional sensitivity analyses revealed a lower VTE proportion with the exclusion of Ren et al.'s data (0.43); this proportion increased with the exclusion of Criel et al.'s study (0.52) (Supplementary 4). All the studies evaluated systematically for the presence of DVT events only (PE was not a primary aim). Hence, this pooled proportion represents the proportion of DVT events and may underestimate the overall VTE proportion.

## The Proportion of VTE Utilizing Non-Systematic Screening

**Figure 4:**
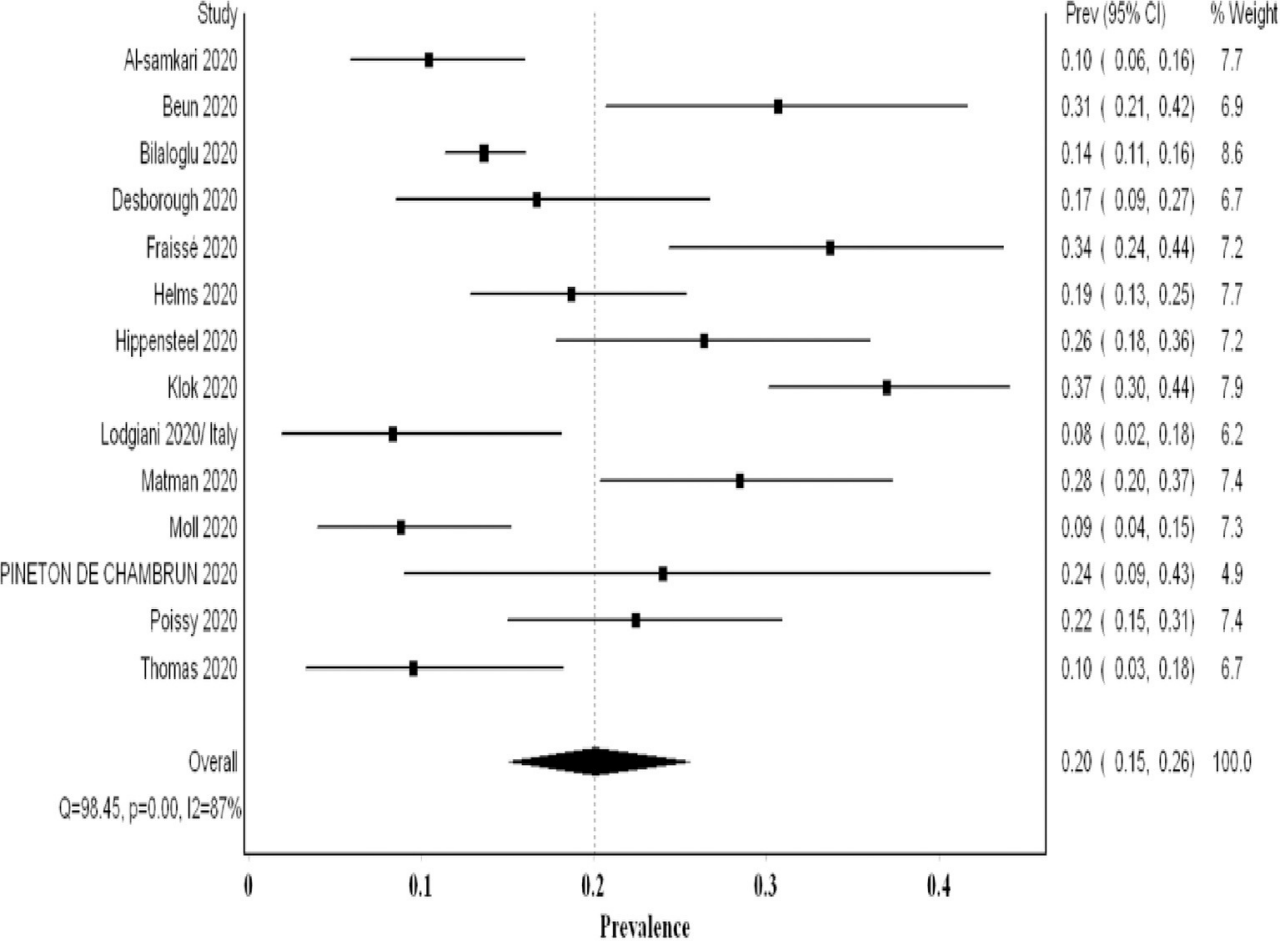
Forest plot showing the pooled proportion of VTE events utilizing non-systematic screening methods.

In most studies utilizing non-systematic screening, the authors addressed the high threshold for screening and imaging due to infection control implications. They stated that this might have underestimated the true prevalence. The analysis of 14 studies examining 2,085 patients revealed a pooled proportion of VTE of 0.20 (95% CI 0.15, 0.26; *I*^2^ 87%; Q 98.4) (Figure 4). The funnel plot suggested a publication bias (Supplementary 5). On sensitivity analysis, the final point estimate did not significantly change with the ordered exclusion of the constituent studies (Supplementary 6).

## The Proportion of DVT Events

**Figure 5:**
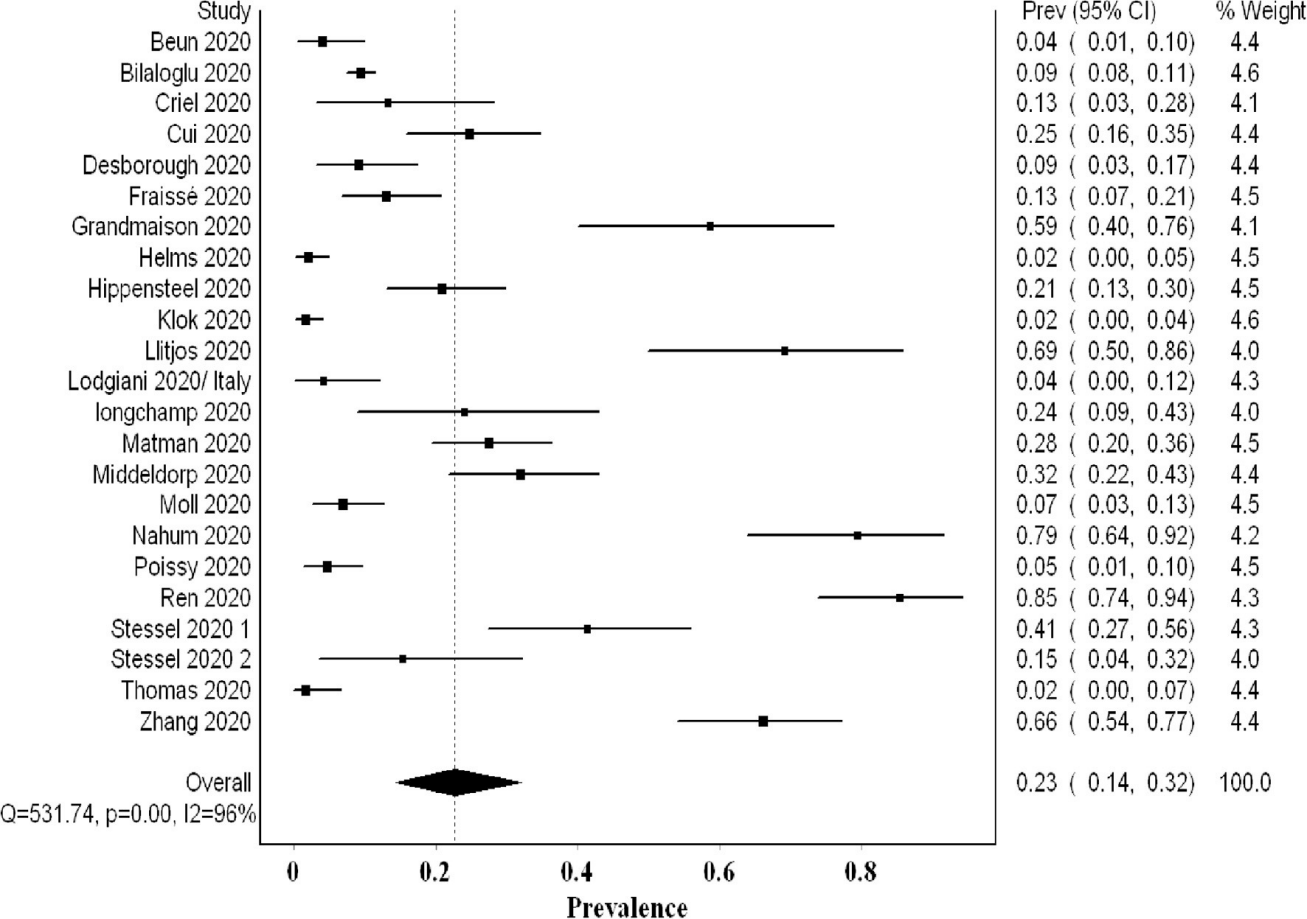
Forest plot showing the overall pooled proportion of DVT.

The overall pooled proportion of DVT from 22 studies examining a total of 2,401 was 0.23 (95% CI 0.14, 0.32; *I*^2^ 96%; Q 531) with significant heterogeneity (Figure 5). The funnel plot suggested a publication bias (Supplementary 7), whereas the sensitivity analysis suggested a consistency of the final point estimate with ordered-single-study exclusion (Supplementary 8). The pooled proportion of DVT from studies utilizing non-systematic screening was 0.08 (95% CI 0.04, 0.12; *I*^2^ 87%; Q 85) (Supplementary 9).

## The Proportion of Pe Events

**Figure 6:**
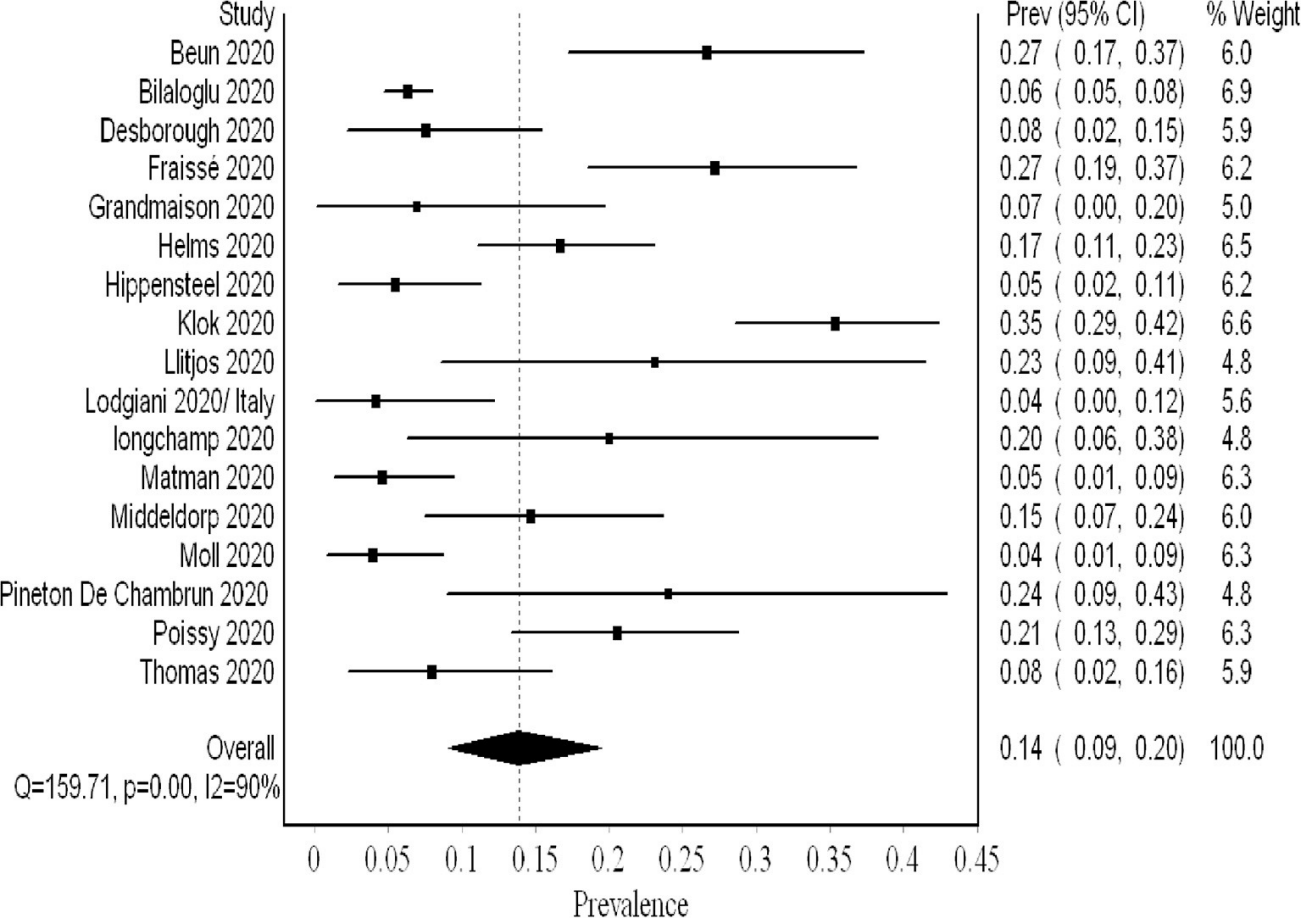
Forest plot showing the overall pooled proportion of PE.

PE was not screened systematically. The analysis of 2,096 patients (17 studies) revealed a pooled proportion of 0.14 (95% CI 0.09, 0.20; *I*^2^ 90%; Q 159) (Figure 6). The funnel plot revealed a major asymmetry suggestive of publication bias (Supplementary 10). Sensitivity analysis showed consistency of the results upon single-study-ordered exclusion.

## Thromboprophylaxis Strategy

**Figure 7:**
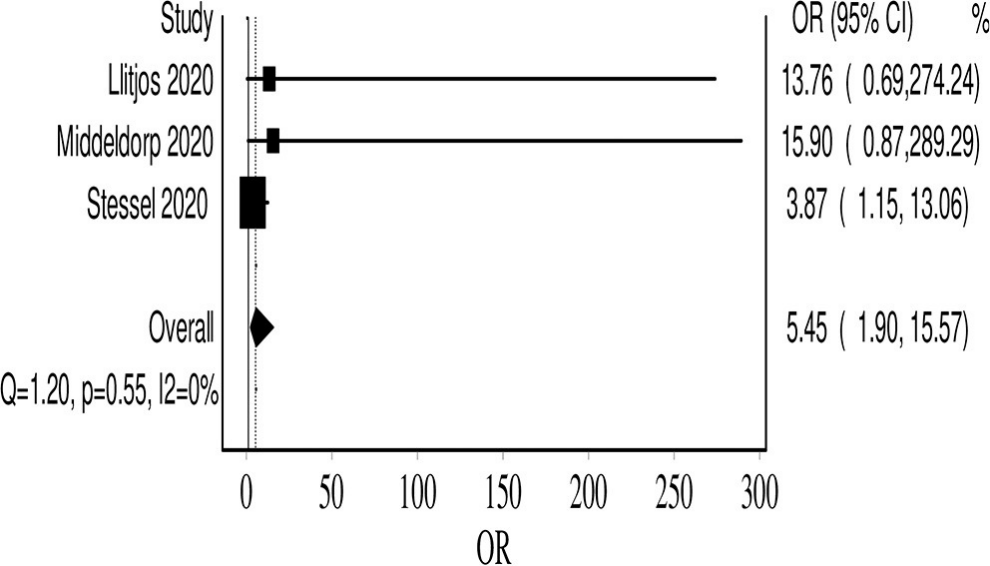
Forest plot showing the VTE event odds in the prophylactic anticoagulation group, compared with therapeutic dosing.

Six studies reported the number of VTE events in patients receiving prophylactic anticoagulation (479 patients) compared with therapeutic dosages (83 patients). The dosages and definitions varied across these studies. In one study (pre- and post-intervention), a higher prophylactic dosage of nadroparin with adjustment guided by factor X-a activity (labeled as semi-therapeutic) was compared with standard prophylactic dose (4, 14, 20). For synthesis, we considered this adjusted dosage therapeutic and analyzed it in the corresponding arm (due to the paucity of studies). The VTE odds ratio (OR) was increased in the prophylactic anticoagulation group with uncertainty in the final point estimate OR 2.34 (95% CI 0.77, 7.14; *I*^2^ 53%; Q 10). Three studies utilized systematic screening; hence, they provided a better estimate of the true VTE prevalence (20). In an exploratory analysis, we analyzed these studies separately, and the results showed significantly increased odds of VTE events with prophylactic dosing OR 5.45 (95% CI 1.90, 15.57; *I*^2^ 0%; Q 1.2), and there was no evidence of heterogeneity (Figure 7).

## Quality Assessment and Risk of Bias Assessment

Most of the constituent studies had a moderate or unclear risk of bias (Table 2). Although the number of included studies is adequate, the funnel plot suggested publication bias (its value is limited in assessing prevalence studies publication bias). There was also reporting bias, as the reporting of distal DVT, PE, and VTE, method of diagnosis, and dosing of chemoprophylaxis varied across studies.

**Table 2:**
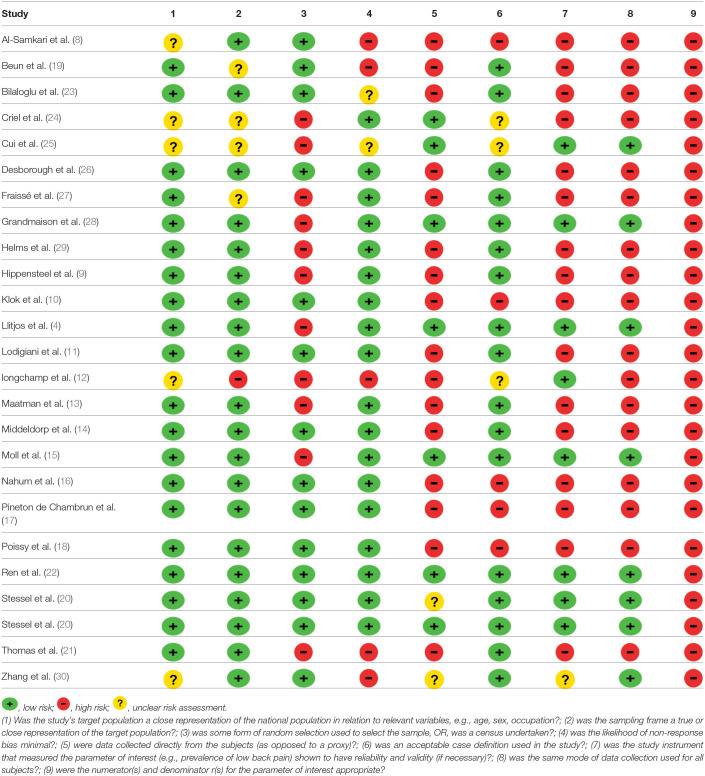
Table summarizing the risk of bias assessment.

## Discussion

Our meta-analysis comprised over 2,500 patients and revealed a high VTE prevalence of 0.31 (95% CI 0.24, 0.39) in critically ill COVID-19 patients. This prevalence increased to 0.48 (95% CI 0.33, 0.63) when systematic screening was utilized, meaning that almost one in two critical COVID-19 patients suffers from VTE. Furthermore, this heightened prevalence of VTE when systematic screening was used did not include PE since it was not part of systematic screening. Hence, screening for PE systematically could have possibly further increased VTE prevalence. Even when non-systematic screening was utilized, VTE prevalence remained high at 0.20 (95% CI 0.15, 0.26). Regarding PE and DVT prevalence, the overall prevalence of DVT (0.23) was higher than that of PE (0.14). This concurs with finding a high prevalence of undiagnosed DVT in an autopsy evaluation of COVID-19 patients (31). Additionally, it may argue against the earlier literature suggesting that PE prevalence was much higher than DVT, proposing that PE events can originate in the lung's vasculature in patients with severe acute respiratory syndrome coronavirus 2 (SARS-CoV-2) infection (32).

Our analysis revealed that approximately 40/100 additional DVTs are detected by systematic screening (0.48) compared with non-systematic screening (0.08). This is likely due to the fact that asymptomatic DVT can be overlooked in non-systematic screening. On the opposite side, PE is more likely to be associated with easily detected signs (sudden deterioration, unexplained tachycardia or sudden changes in the ventilator settings) especially in the context of the ICU.

A recent study by Zhang et al. evaluated the utility of bedside ultrasonography in the diagnosis of DVT. It revealed a significantly higher DVT prevalence in deceased patients than in surviving COVID-19 critically ill patients [94% (33/35) vs. 47% (22/46), *P* < 0.001] (30). Moreover, Wichmann et al. analyzed autopsies of 12 COVID-19 patients. They found that 7 (58%) had undiagnosed VTE, whereas in 4 (33.3%), massive PE was the direct cause of death (31). Based on these data, we understand that the high mortality reported by many studies may actually be attributed to undiagnosed fatal VTE events. Consequently, studies with high mortality will likely underestimate the true VTE prevalence when deceased patients are excluded from screening. We additionally understand the impact of prevention and early identification on patient's morbidity and mortality.

Tang et al. showed that prophylactic dosing of heparin in high-risk COVID-19 patients is associated with significantly lower mortality (33). This led the International Society on Thrombosis and Hemostasis (ISTH) among other societies to recommend a prophylactic dosage of pharmacological anticoagulants (LMWH or fondaparinux) for all hospitalized COVID-19 patients (3, 34). However, it seemed that prophylactic anticoagulation is not sufficient for severe COVID-19 patients. This was concluded in a study by Llitjos et al. where they found a higher prevalence of VTE in patients on a prophylactic dose of anticoagulation (100%) compared with therapeutic anticoagulation (56%) (4). More recently, Stessel et al. attempted the first quasi-experimental trial (pre- and post-intervention) comparing the mortality and incidence of VTE between conventional prophylaxis (once-daily nadroparin calcium 2,850 IU) compared with an individualized semi-therapeutic, prophylactic dosage guided by factor Xa activity (semi-therapeutic dosing). Both mortality (3.8 vs. 39.1%, *P* < 0.001) and VTE (15.3 vs. 41.3%, *P* = 0.03) were significantly lower in the aggressive thromboprophylaxis group (20). Emerging evidence showed that even in COVID-19 patients receiving therapeutic anticoagulation, there is a high incidence of heparin resistance and sub-optimal peak in anti-Xa levels (19, 35). This may explain, in part, the high rate of VTE in patients on usual prophylactic doses and even in patients on therapeutic dosing (although relatively at a lower rate).

Our review also aimed to address the uncertainty of using higher vs. standard prophylactic doses. In an exploratory manner, we limited our analysis to studies that only used systematic screening and thus reduce the chances of missing fatal VTE events; we found that prophylactic dosing was associated with increased odds of VTE compared with therapeutic dosing (one study was counted in the therapeutic side although it used subtherapeutic dosing, due to limited studies) (20). The results were homogenous. The reader should consider that the odds of VTE in the therapeutic arm were lower even in the likely event that those patients may have had VTE predisposing conditions, for which they were initiated on this therapeutic dosing (except Stessel et al.'s study, which was protocolized). This small exploratory unadjusted comparison suggests a value for a higher dosing or therapeutic chemoprophylaxis. Nonetheless, this will be ascertained by a number of ongoing trials aiming to address the efficacy and safety of various chemoprophylactic dosages (prophylactic, intermediates, weight-adjusted, or therapeutic); examples of such trials are IMPROVE (http://www.clinicaltrials.gov, NCT04367831), COVI-DOSE (http://www.clinicaltrials.gov, NCT04373707), and Hep-COVID (https:www.clinicaltrials.gov, NCT04401293). The safety of intensive thromboprophylaxis was not addressed in our review due to data paucity. Nonetheless, two recent observational studies suggested that this intensive thromboprophylaxis is safe in terms of inducing major bleeding events (36, 37). Thus, we believe that the intensive thromboprophylaxis protocol suggested by Stessel et al. seems promising as a chemoprophylaxis regimen until further data from ongoing randomized clinical trials (RCTs) become available (20).

Limitations of our review are the heterogeneity in the pooled prevalence in the constituent studies. This is likely due to varying detection methods (systematic vs. non-systematic, imaging modalities used, timing, etc.), screening threshold (many studies reported that the threshold was high due to infection control concerns), varying severity of illness, prophylaxis strategies, and dosage, missing VTE in deceased patients of fatal VTE events, and varying and insufficient follow-ups. Additionally, the inability to provide a mortality comparison between the VTE group and the non-VTE group due to data paucity (we contacted the primary authors; however, we could not get the data necessary for its computation) and limited conclusion provided by the comparison of VTE in the therapeutic vs. prophylactic anticoagulation groups (small number of studies, absence of adjustment, and varying doses between studies). Moreover, the retrospective nature of the included studies, inability to accurately compute the prevalence of PE (absence of systematic PE screening), and absence of autopsies to ascertain causes of death add to the limitations of our review.

Notwithstanding this, there are many strengths to our review that are worthy of mention. This is the most extensive review examining the prevalence of VTE exclusively in critically ill patients. Additionally, the review examines VTE prevalence based on the utilized screening method providing the readers with a better estimate of VTE prevalence. We also pooled a proportion that reflects the prevalence; nonetheless, we acknowledged its limited accuracy. Finally, the results of the limited comparison between lower and higher dosing of chemoprophylaxis may help inform therapeutic decisions until further data from RCTs become available.

Future research direction should evaluate the utility of systematic screening and early therapeutic anticoagulation dosage on outcomes (VTE progression, ICU stay, and mortality). The utility of systematic screening with US at regular intervals to ascertain the exact prevalence of VTE is needed. In these studies, patients with distal DVT should be temporally followed up and compared with a non-DVT cohort to determine the incidence of proximal DVT, PE, and mortality events. This will ascertain the exact need for therapy in these patients.

In conclusion, our review of critically ill COVID-19 patients revealed a high prevalence of VTE events. This prevalence is higher when systematic screening is utilized. Our review suggested a potential for higher prophylactic or therapeutic dosages in reducing VTE burden. Data from ongoing RCTs are awaited to further confirm the findings of our review.

## Data Availability Statement

The original contributions presented in the study are included in the article/Supplementary Materials, further inquiries can be directed to the corresponding author/s.

## Author Contributions

MM conceived the idea of the review and formed the team, performed the analysis, constructed the tables and figures, and wrote the initial draft. MM conducted the initial search and with SM conducted the screening. MM, KS, SA-S, SM, SI, MN, and LA extracted the data. The manuscript was then critically reviewed and revised by all the study authors. The final version was approved by all authors for publication.

## Conflict of Interest

The authors declare that the research was conducted in the absence of any commercial or financial relationships that could be construed as a potential conflict of interest.
