# Supplementary material for: Prevalence of Venous Thromboembolism in Critically Ill COVID-19 Patients: Systematic Review and Meta-Analysis
Source: Front Cardiovasc Med. 2021 Jan 8;7:598846. doi: 10.3389/fcvm.2020.598846 (PMC7874113; doi:10.3389/fcvm.2020.598846)

**S1:** Funnel plot showing significant asymmetry suggestive of publication bias


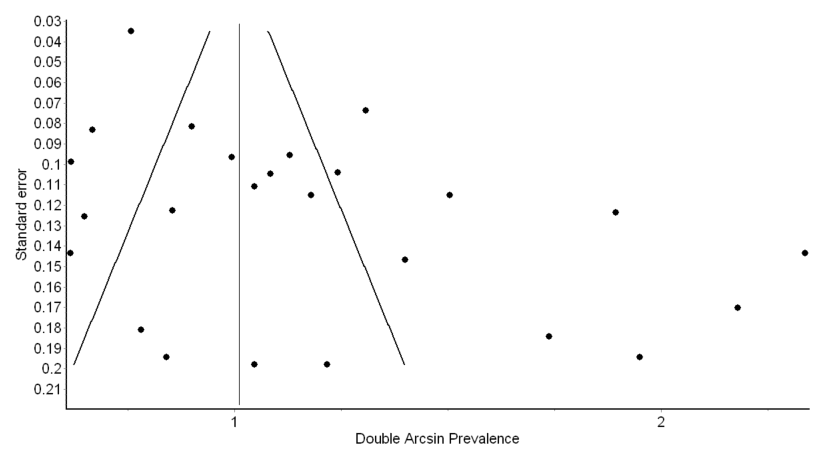


**S2:** Sensitivity analysis results of the primary outcome of VTE events, showing no significant resultant change with the consequent exclusion of the constituent studies.

| **Excluded study** | **Pooled Prevalence** | **LCI 95%** | **HCI 95%** | **Cochran Q** | **p** | **I 2** | **I 2 LCI 95%** | **I 2 HCI 95%** |
| --- | --- | --- | --- | --- | --- | --- | --- | --- |
| Al-samkari 2020 | 0.322552 | 0.245101 | 0.405154 | 364.9055 | 0 | 93.697 | 91.76977 | 95.17294 |
| Beun 2020 | 0.311864 | 0.234945 | 0.394293 | 380.8379 | 0 | 93.96068 | 92.14117 | 95.35893 |
| Bilaloglu 2020 | 0.32105 | 0.242205 | 0.4053 | 304.9319 | 0 | 92.45733 | 90.00381 | 94.30865 |
| Criel 2020 | 0.318958 | 0.242324 | 0.400773 | 381.4261 | 0 | 93.97 | 92.15426 | 95.36552 |
| Cui 2020 | 0.314552 | 0.237101 | 0.397479 | 382.9414 | 0 | 93.99386 | 92.18779 | 95.38239 |
| Desborough 2020 | 0.31831 | 0.240978 | 0.400946 | 381.3885 | 0 | 93.9694 | 92.15343 | 95.3651 |
| Fraissé 2020 | 0.310587 | 0.233746 | 0.392979 | 377.8954 | 0 | 93.91366 | 92.07505 | 95.32571 |
| Grandmaison 2020 | 0.30178 | 0.227915 | 0.381111 | 367.2429 | 0 | 93.73711 | 91.82637 | 95.20119 |
| Helms 2020 | 0.317816 | 0.239055 | 0.402105 | 381.0644 | 0 | 93.96428 | 92.14622 | 95.36147 |
| Hippensteel 2020 | 0.313827 | 0.236293 | 0.396879 | 382.5353 | 0 | 93.98748 | 92.17883 | 95.37788 |
| Klok 2020 | 0.309194 | 0.232109 | 0.391926 | 365.42 | 0 | 93.70587 | 91.78229 | 95.17919 |
| Llitjos 2020 | 0.298286 | 0.225533 | 0.376475 | 359.3913 | 0 | 93.60029 | 91.63316 | 95.10493 |
| Lodgiani 2020/ Italy | 0.322641 | 0.245899 | 0.404434 | 375.3222 | 0 | 93.87193 | 92.01634 | 95.29624 |
| longchamp 2020 | 0.311131 | 0.235209 | 0.392445 | 381.9555 | 0 | 93.97836 | 92.16601 | 95.37143 |
| Matman 2020 | 0.312944 | 0.235268 | 0.396196 | 381.4387 | 0 | 93.9702 | 92.15454 | 95.36566 |
| Middeldorp 2020 | 0.305216 | 0.230452 | 0.385442 | 364.0339 | 0 | 93.68191 | 91.74846 | 95.16232 |
| Moll 2020 | 0.323337 | 0.246391 | 0.405335 | 366.5302 | 0 | 93.72494 | 91.80919 | 95.19261 |
| Nahum 2020 | 0.293362 | 0.223155 | 0.368807 | 335.2661 | 0 | 93.13978 | 90.97992 | 94.78246 |
| PINETON DE CHAMBRUN 2020 | 0.314095 | 0.237832 | 0.395682 | 383.0211 | 0 | 93.99511 | 92.18954 | 95.38328 |
| Poissy 2020 | 0.315752 | 0.237727 | 0.399286 | 383.0201 | 0 | 93.99509 | 92.18952 | 95.38327 |
| Ren 2020 | 0.289019 | 0.222849 | 0.359975 | 295.9326 | 0 | 92.22796 | 89.67367 | 94.15043 |
| Stessel 2020 1 | 0.307578 | 0.232038 | 0.388599 | 375.8785 | 0 | 93.881 | 92.0291 | 95.30264 |
| Stessel 2020 2 | 0.317731 | 0.241218 | 0.399458 | 382.2769 | 0 | 93.98342 | 92.17312 | 95.37501 |
| Thomas 2020 | 0.322311 | 0.245377 | 0.404335 | 374.516 | 0 | 93.85874 | 91.99777 | 95.28693 |

**S3:** Funnel plot of studies evaluating VTE events via systematic screening, and showing significant asymmetry suggestive of publication bias


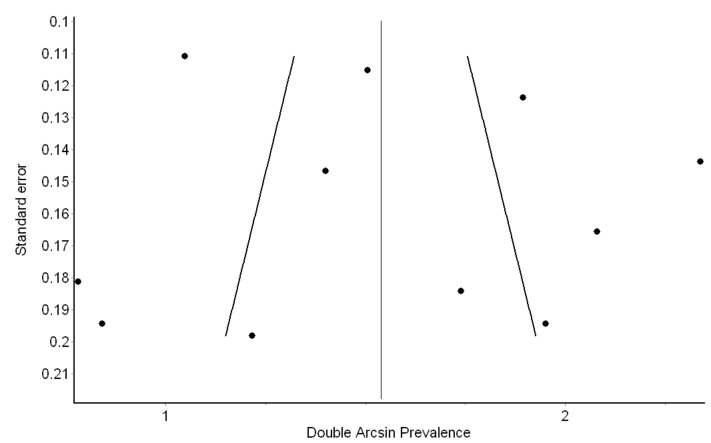


**S4:** Sensitivity analysis results of the studies evaluating VTE events utilizing systematic screening, and showing no significant resultant change with the consequent exclusion of the constituent studies.

| **Excluded study** | **Pooled Prevalence** | **LCI 95%** | **HCI 95%** | **Cochran Q** | **p** | **I 2** | **I 2 LCI 95%** | **I 2 HCI 95%** |
| --- | --- | --- | --- | --- | --- | --- | --- | --- |
| Criel 2020 | 0.516473 | 0.367303 | 0.664219 | 90.74551 | 1.11E-15 | 90.08215 | 83.90028 | 93.89035 |
| Cui 2020 | 0.50566 | 0.352612 | 0.65819 | 85.85734 | 1.10E-14 | 89.51749 | 82.85111 | 93.59242 |
| Grandmaison 2020 | 0.468612 | 0.308549 | 0.631891 | 108.1384 | 0 | 91.67733 | 86.81062 | 94.74829 |
| Llitjos 2020 | 0.458549 | 0.302437 | 0.618739 | 104.6328 | 0 | 91.39849 | 86.30787 | 94.59646 |
| longchamp 2020 | 0.494234 | 0.334572 | 0.654473 | 106.6245 | 0 | 91.55916 | 86.59788 | 94.68385 |
| Middeldorp 2020 | 0.480065 | 0.309794 | 0.652621 | 109.2989 | 0 | 91.7657 | 86.96938 | 94.79658 |
| Nahum 2020 | 0.451262 | 0.29883 | 0.608301 | 97.85724 | 0 | 90.80293 | 85.22543 | 94.27488 |
| Ren 2020 | 0.437462 | 0.302832 | 0.576771 | 74.9701 | 1.60E-12 | 87.99521 | 79.97728 | 92.80243 |
| Stessel 2020 1 | 0.485683 | 0.320901 | 0.651997 | 108.4135 | 0 | 91.69845 | 86.84859 | 94.75982 |
| Stessel 2020 2 | 0.512764 | 0.360477 | 0.663901 | 95.74656 | 1.11E-16 | 90.60018 | 84.85433 | 94.16622 |
| Zhang 2020 | 0.459413 | 0.299795 | 0.623201 | 99.79202 | 0 | 90.98124 | 85.55073 | 94.37079 |

**S5:** Funnel plot of studies evaluating VTE events via non-systematic screening, and showing significant asymmetry suggestive of publication bias


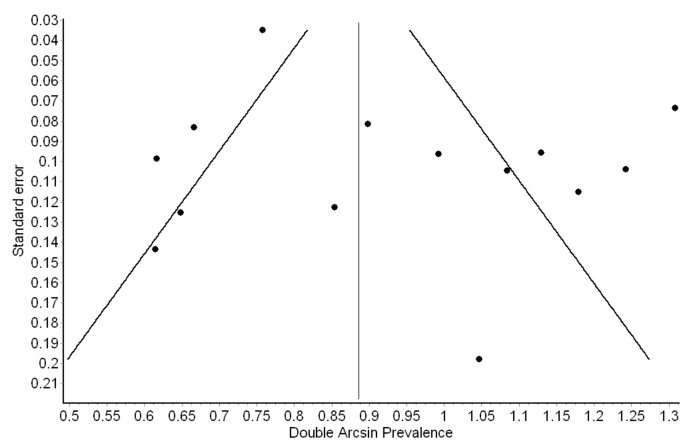


**S6:** Sensitivity analysis results of the studies evaluating VTE events utilizing non-systematic screening, and showing no significant resultant change with the consequent exclusion of the constituent studies.

| **Excluded study** | **Pooled Prevalence** | **LCI 95%** | **HCI 95%** | **Cochran Q** | **p** | **I ^2^** | **I 2 LCI 95%** | **I 2 HCI 95%** |
| --- | --- | --- | --- | --- | --- | --- | --- | --- |
| **Al-samkari 2020** | 0.209467 | 0.156241 | 0.268131 | 90.97259 | 3.20E-14 | 86.80921 | 79.17325 | 91.64551 |
| **Beun 2020** | 0.193138 | 0.142701 | 0.249092 | 91.69405 | 2.32E-14 | 86.913 | 79.35813 | 91.70281 |
| **Bilaloglu 2020** | 0.206847 | 0.152042 | 0.267549 | 75.9356 | 2.44E-11 | 84.19713 | 74.45202 | 90.22504 |
| **Desborough 2020** | 0.2027 | 0.149721 | 0.261352 | 98.38258 | 1.11E-15 | 87.80272 | 80.93391 | 92.19695 |
| **Fraissé 2020** | 0.190956 | 0.141868 | 0.245347 | 86.13217 | 2.76E-13 | 86.06792 | 77.84654 | 91.23826 |
| **Helms 2020** | 0.201428 | 0.147066 | 0.261836 | 98.42174 | 1.11E-15 | 87.80757 | 80.94246 | 92.19966 |
| **Hippensteel 2020** | 0.195697 | 0.143935 | 0.253165 | 94.66625 | 6.11E-15 | 87.32389 | 80.0879 | 91.93034 |
| **Klok 2020** | 0.18761 | 0.144315 | 0.235095 | 62.28354 | 8.63E-09 | 80.73327 | 68.00874 | 88.39662 |
| **Lodgiani 2020/ Italy** | 0.209052 | 0.156685 | 0.266692 | 94.81275 | 5.77E-15 | 87.34347 | 80.1226 | 91.94122 |
| **Matman 2020** | 0.194142 | 0.143015 | 0.250898 | 91.58551 | 2.43E-14 | 86.89749 | 79.33052 | 91.69424 |
| **Moll 2020** | 0.210665 | 0.158104 | 0.268474 | 90.67373 | 3.65E-14 | 86.76574 | 79.09574 | 91.62153 |
| **PINETON DE CHAMBRUN 2020** | 0.198088 | 0.146839 | 0.254821 | 97.78051 | 1.55E-15 | 87.72762 | 80.80154 | 92.15503 |
| **Poissy 2020** | 0.198431 | 0.14546 | 0.257264 | 97.14639 | 2.00E-15 | 87.64751 | 80.66021 | 92.11036 |

**S7:** Funnel plot of studies evaluating DVT events, showing significant asymmetry suggestive of publication bias


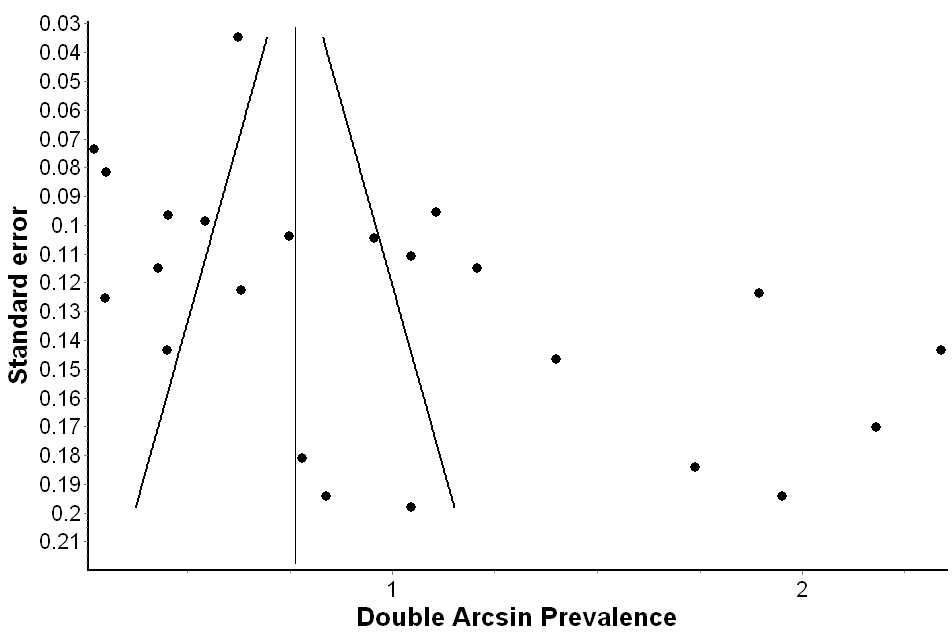


**S8:** Sensitivity analysis results of the studies evaluating VTE events utilizing non-systematic screening, and showing no significant resultant change with the consequent exclusion of the constituent studies.

| **Excluded study** | **Pooled Prevalence** | **LCI 95%** | **HCI 95%** | **Cochran Q** | **p** | **I 2** | **I 2 LCI 95%** | **I 2 HCI 95%** |
| --- | --- | --- | --- | --- | --- | --- | --- | --- |
| Beun 2020 | 0.237617 | 0.150324 | 0.337314 | 523.0904 | 0 | 95.9854 | 94.88151 | 96.85121 |
| Bilaloglu 2020 | 0.234298 | 0.137088 | 0.347481 | 507.1854 | 0 | 95.8595 | 94.70857 | 96.7601 |
| Criel 2020 | 0.230342 | 0.144616 | 0.328684 | 531.7287 | 0 | 96.05062 | 94.97091 | 96.89852 |
| Cui 2020 | 0.225404 | 0.139779 | 0.3241 | 525.0014 | 0 | 96.00001 | 94.90156 | 96.8618 |
| Desborough 2020 | 0.233561 | 0.146537 | 0.333292 | 530.554 | 0 | 96.04187 | 94.95893 | 96.89217 |
| Fraissé 2020 | 0.231348 | 0.14401 | 0.331709 | 531.718 | 0 | 96.05054 | 94.9708 | 96.89846 |
| Grandmaison 2020 | 0.212944 | 0.132171 | 0.306443 | 503.4172 | 0 | 95.82851 | 94.66592 | 96.73771 |
| Helms 2020 | 0.240506 | 0.153137 | 0.340034 | 497.8234 | 0 | 95.78164 | 94.60136 | 96.70388 |
| Hippensteel 2020 | 0.227216 | 0.140835 | 0.326729 | 528.222 | 0 | 96.0244 | 94.93499 | 96.87949 |
| Klok 2020 | 0.241174 | 0.154133 | 0.340219 | 483.9082 | 0 | 95.66033 | 94.43396 | 96.6165 |
| Llitjos 2020 | 0.209414 | 0.130001 | 0.30145 | 494.0505 | 0 | 95.74942 | 94.55695 | 96.68065 |
| Lodgiani 2020/ Italy | 0.236857 | 0.150011 | 0.336044 | 526.9466 | 0 | 96.01478 | 94.9218 | 96.87251 |
| longchamp 2020 | 0.225477 | 0.140825 | 0.322881 | 529.6863 | 0 | 96.03539 | 94.95005 | 96.88747 |
| Matman 2020 | 0.224156 | 0.138655 | 0.322814 | 518.1212 | 0 | 95.94689 | 94.82868 | 96.82332 |
| Middeldorp 2020 | 0.22222 | 0.13789 | 0.319521 | 516.4412 | 0 | 95.93371 | 94.81057 | 96.81377 |
| Moll 2020 | 0.235486 | 0.147655 | 0.336086 | 526.6313 | 0 | 96.01239 | 94.91853 | 96.87078 |
| Nahum 2020 | 0.204641 | 0.128237 | 0.293126 | 461.6674 | 0 | 95.45127 | 94.14442 | 96.46646 |
| Poissy 2020 | 0.237342 | 0.149551 | 0.337714 | 520.9411 | 0 | 95.96883 | 94.85879 | 96.83921 |
| Ren 2020 | 0.200647 | 0.128671 | 0.283612 | 409.1946 | 0 | 94.86797 | 93.32992 | 96.05136 |
| Stessel 2020 1 | 0.218678 | 0.13586 | 0.314327 | 512.6093 | 0 | 95.90331 | 94.76881 | 96.79177 |
| Stessel 2020 2 | 0.229197 | 0.143777 | 0.327248 | 531.5845 | 0 | 96.04955 | 94.96944 | 96.89774 |
| Thomas 2020 | 0.240022 | 0.153038 | 0.339097 | 517.8454 | 0 | 95.94474 | 94.82571 | 96.82175 |
| Zhang 2020 | 0.208665 | 0.132167 | 0.296893 | 445.7522 | 0 | 95.28886 | 93.9186 | 96.35037 |

**S9:** Forest plot showing the pooled proportion of DVT derived from studies utilizing non-systematic screening


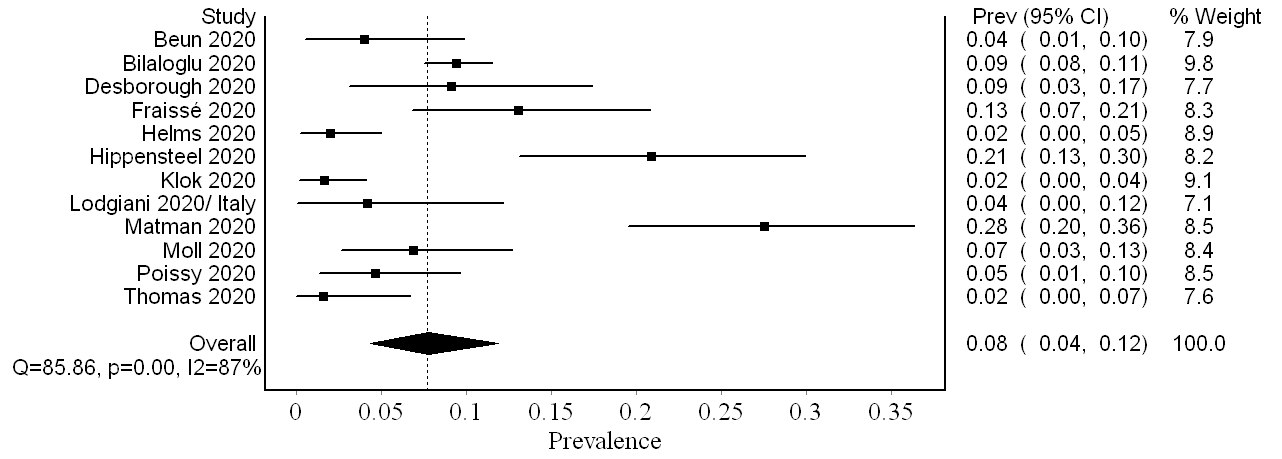


**S10:** Funnel plot of studies evaluating the proportion of PE events, showing major asymmetry suggestive of a publication bias


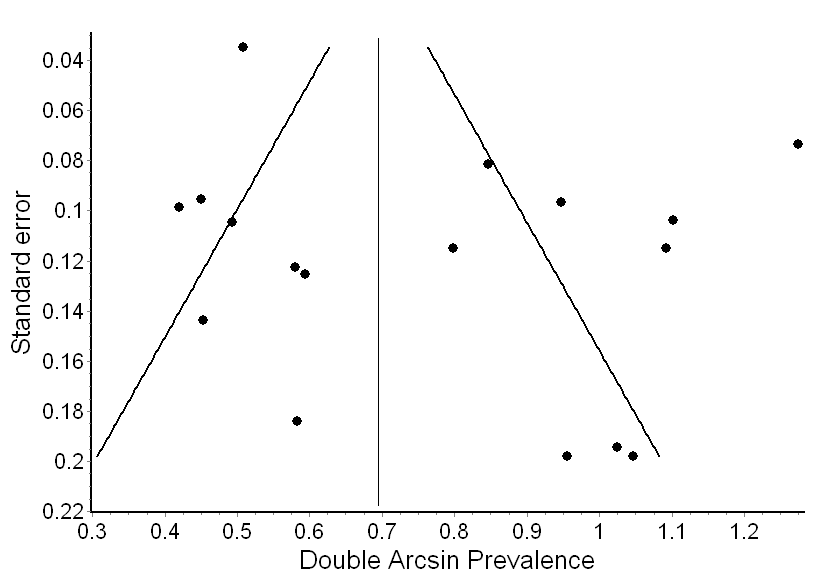

Supplement: Supplementary file 1 [file Data_Sheet_1.docx]
